# A Broadband MS-Based Circularly Polarized Antenna Array Using Sequential-Phase Feeding Network

**DOI:** 10.3390/mi15081051

**Published:** 2024-08-20

**Authors:** Hung Pham-Duy, Dat Nguyen-Tien, Thanh Nguyen-Ngoc, Duc-Nguyen Tran-Viet, Thai Nguyen-Dinh, Tuyen Danh Pham, Hung Tran-Huy

**Affiliations:** 1 Faculty of Electrical and Electronic Engineering, PHENIKAA University, Yen Nghia, Ha Dong, Hanoi 12116, Vietnam; hung.phamduy@phenikaa-uni.edu.vn (H.P.-D.); dat.nguyentien@phenikaa-uni.edu.vn (D.N.-T.); thanh.nguyenngoc@phenikaa-uni.edu.vn (T.N.-N.); 2 Faculty of Radio-Electronic Engineering, Le Quy Don Technical University, Hanoi 11917, Vietnam; tranvietducnguyen@lqdtu.edu.vn (D.-N.T.-V.); thaind@lqdtu.edu.vn (T.N.-D.); 3 IT Department, FPT University, Greenwich Vietnam, Hanoi Campus, Hanoi 11311, Vietnam; tuyenpd5@fe.edu.vn

**Keywords:** circular polarization, microstrip patch, metasurface, broadband, sequential-phase feeding structure

## Abstract

This paper introduces the design of a circularly polarized metasurface-based antenna array for C-band satellite applications that owns broadband operation and high gain characteristics. The single radiating element comprises a Y-shape patch and an above-placed 2 × 2 unit-cell metasurface. Further improvement in operating bandwidth and broadside gain is achieved by arranging four single elements in a 2 × 2 configuration and a sequential-phase feed network. A prototype has been fabricated and measured to validate the feasibility of the proposed antenna array. The measured operating bandwidth is 20% (4.50–5.50 GHz), which is an overlap between a −10 dB impedance bandwidth of 29.8% (4.50–5.99 GHz) and a 3 dB axial ratio bandwidth of 20% (4.50–5.50 GHz). Across this operating band, the peak broadside gain is 10.5 dBi. Compared with the recently published studies, the proposed array is a prominent design for producing a wide operating bandwidth and relatively high gains while maintaining the overall compact dimensions.

## 1. Introduction

In the past few years, satellite communications have becoming more and more popular since they offer a wide range of applications such as television broadcasting, navigation, weather forecast, and disaster management. For the rising demands of miniaturizing the satellite structures to be applied in more detailed missions, the circularly polarized (CP) printed antenna is a promising candidate due to its characteristics, which include the ability to counter multi-path distortions and polarization mismatch losses [1,2]. Nevertheless, one issue that may deter CP microstrip patch antennas from being deployed on satellites is their limited bandwidth (BW), which is constrained to around 3%, as reported in the literature. Consequently, there is an urgent need to extend the CP operation BW of that antenna type.

Recently, there have been a large number of studies focusing on improving the BW for printed antennas with CP radiation. A recent proposal in [3] has employed two pairs of loaded stubs and four shorting pins to a conventional truncated radiator for an implantable antenna, which produces a broad operating band of 19%. The prototypes in [4,5,6] managed to make changes in the ground planes by using artificial ground structures with different shapes or introducing a T-slot into the metallic ground (DGS). Other approaches include the use of end-fire configuration [7], the combination of metallic walls and shorting pins [8], metamaterial reflectors [9], and metasurfaces (MSs) [10,11]. Although those configurations have achieved wideband properties [4,10,11], they still suffer from the drawbacks of either large sizes [5,8,9] or high-profile configurations [4,5,7,9]. One of the most noticeable disadvantages of these designs is that they produce low peak gains of less than 10 dBi. Employing parasitic elements [12,13,14] is also an effective way to extend the operating frequency range. However, while the design in [12] produces a very large array of 8 × 8 elements, the latter ones do not surpass the peak gains of 10 dBi.

It is worth noting that using sequential-phase (SP) feeding networks to excite linearly polarized (LP) radiators is an alternative solution to the problem of designing CP-printed antennas, as reported in [15,16,17,18,19,20,21,22]. This technique has been investigated widely because it would provide the designated antennas with low profile, broadband, and high gain properties. Generally, the antennas with the presence of SP feeding structures are fed by only one input port. The elements in the array would then be excited by a network of microstrip lines with various dimensions, which ensures that the phase shift between two adjacent radiators is stable at 90°. The proposals in [15,16] have significantly extended the operating BW despite their relatively low peak gains, which are less than 10 dBi. Although the relatively high maximum gains could be seen in [17,18,19,20,21], they still suffer from large overall sizes. To conclude, it is challenging for antenna researchers to conceptualize a CP patch antenna with broadband operation, compact dimensions, and a high peak gain of over 10 dBi.

This paper introduces an MS-based SP-fed CP antenna that has broadband operation and high gain characteristics. The antenna that produces left-hand circular polarization (LHCP) radiation consists of two sheets of Taconic RF-35 dielectric substrate in which the feeding structure and the ground plane are printed in the lower layer. At the same time, the upper one is composed of four arrays of 2 × 2 MS structures. In comparison with the related works, the proposed antenna is the most compact design. In fact, miniaturizing the antenna while keeping wideband operation is quite difficult. Meanwhile, the operating BW and gain of the proposed antenna are comparable with other related works. This is the main contribution of the proposed design.

## 2. Single Array Element

**Figure 1:**
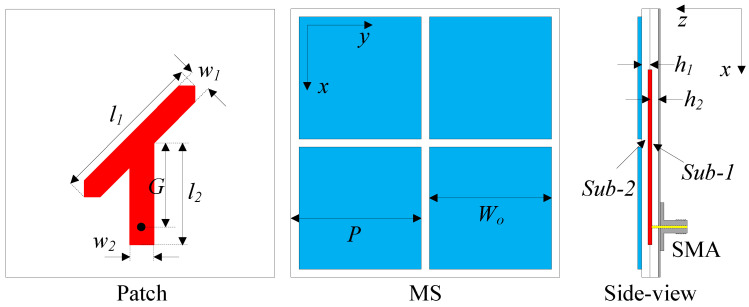
Configuration of the proposed single-element antenna.

Figure 1 illustrates the primary configuration of a CP patch antenna that would further be employed as the radiating element of the proposed array in this paper. The single-element antenna is introduced on two square layers of Taconic RF-35 dielectric substrates with a permittivity of 3.5 and loss tangent of 0.0018. The overall thickness of the design is 3.04 mm, in which each layer occupies the same thickness of $h_{1}$ = $h_{2}$ = 1.52 mm. The ground plane and a modified Y-shaped radiator are placed on two planes of Sub-1, while the 2 × 2 MS structure is introduced on the top side of Sub-2. The excitation to the antenna is fed via a 50-Ω SMA connector. The dimensions of the proposed antenna are as follows: *W* = 24, $l_{1}$ = 9.33, $l_{2}$ = 10, *G* = 9.5, $w_{1}$ = 2, $w_{2}$ = 3, $W_{0}$ = 11.3, *P* = 11.65 (unit: mm).

**Figure 2:**
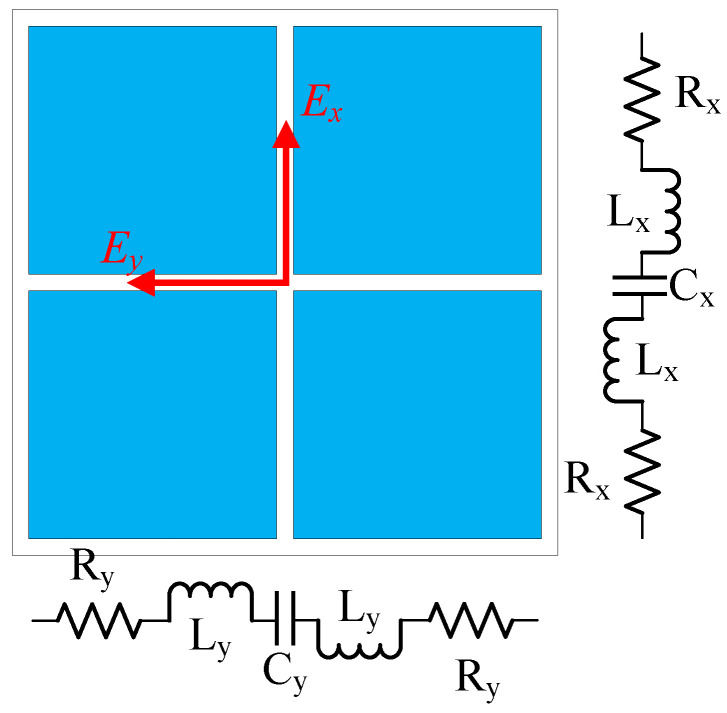
Equivalent circuits of the proposed MS structure.

The literature has reported that antennas with MS structures have been investigated and developed extensively in recent years for applications requiring broadband, compact sizes, and CP operation properties. In such kinds of antenna, the MS plays an essential role as it is the primary radiating element. Eventually, the characteristics of the antenna are significantly influenced by this particular surface. The proposed single CP antenna employs the symmetrical MS structure whose equivalent circuits are depicted in Figure 2. As observed, the circuit elements toward *x*- and *y*-directions are $Z_{x}$ and $Z_{y}$, respectively, with $Z_{x}$ = $Z_{y}$. To achieve CP operation, the MS should be excited by a CP source consisting of two fields, $E_{x}$ and $E_{y}$, with the same magnitude and perpendicular phases. In the proposed design, the Y-shaped patch is used as the primary CP excitation. It is noted that the primary radiating element is the MS layer, which determines the resonant frequency of the antenna. Meanwhile, the Y-shaped patch acts as the CP source, which can produce two orthogonal fields based on its asymmetrical geometry. When combining with the MS, these orthogonal fields can be controlled to achieve equal magnitude and a 90° phase difference. Regarding the size of the MS, the periodicity (*P*) and the unit cell ($W_{0}$) can be adjusted freely as long as the overall dimension is around a half-wavelength at the desired operating band. In the proposed design, the MS has 2 × 2 unit cells with the sizes of the single unit cell and a periodicity of about 11.3 and 11.65 mm. These values ensure that the utilized MS has an overall size of about a half-wavelength at 5.0 GHz. The dominant operating frequency of the proposed MS can be verified by using Characteristic Mode Analysis [23,24].

**Figure 3:**
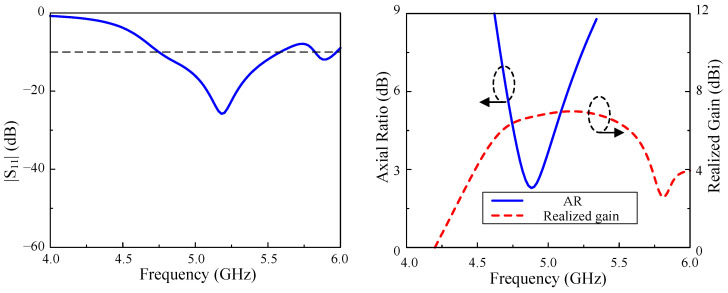
Simulation results regarding S-parameter, axial ratio and realized gain of the single array element.

The simulation results regarding the reflection coefficient, axial ratio, and realized broadside gain of the proposed single-element antenna are illustrated in Figure 3. It is worth noting that the impedance BW of the single element design is relatively large, from 4.75 to 5.59 GHz, which is equivalent to a fractional BW of 16.2% centering at 5.2 GHz. Nevertheless, the overlapped AR BW is quite narrow, at around 2.6% from 4.82 to 4.96 GHz. Within the AR BW, the broadside gains are from 6.5 to 6.8 dBi. To sum up, the narrow CP operation BW and low gain radiation of the single antenna are noticeable drawbacks.

## 3. 2 × 2 Antenna Array

**Figure 4:**
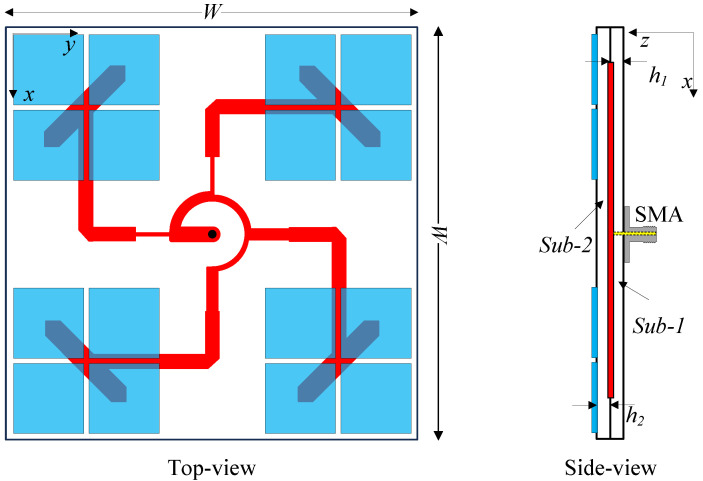
Configuration of the proposed antenna.

To overcome the limitation of both the operating BW and further expand the overall realized gains that the single element antenna possesses, an array of four radiating elements fed by a sequential rotation network and four structures of 2 × 2 unit-cell MS is proposed, as illustrated in Figure 4. The principle for these improvements is thoroughly investigated in [19,25]. The proposed array consists of two sheets of Taconic RF-35 dielectric substrate, which are denoted as Sub-1 and Sub-2. The radiating elements, the feeding structures and the ground plane are printed on two faces of the bottom substrate. The MS structures are introduced on the top layer of Sub-2, which are corresponding to the radiators located below. Each layer has a width of *W* = 61 mm and a thickness of $h_{1}$ = $h_{2}$ = 1.52 mm.

**Figure 5:**
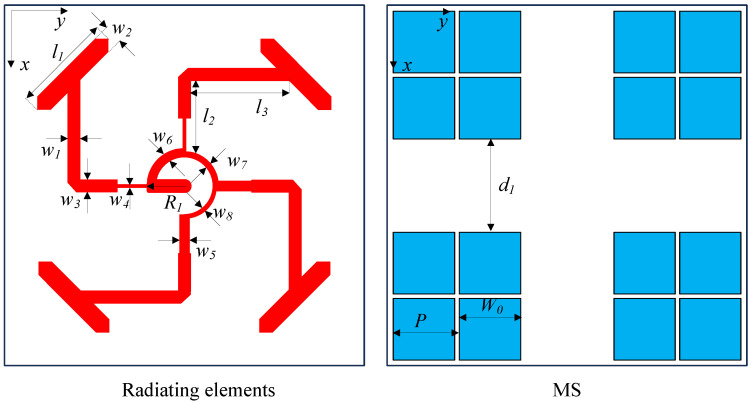
Geometry of the radiating elements and the metasurface.

The detailed geometries of the proposed array regarding the radiating elements, the feeding structure and the MS are exhibited in Figure 5. It is worth noting that the feeding structure plays the most important role in the CP array designs. The array is fed at the center with a 50-Ω SMA connector. Four elements are fed with equal magnitude and 90° phase difference. The optimized dimensions are as follows: $w_{1}$ = 2, $w_{2}$ = 2, $w_{3}$ = 2, $w_{4}$ = 0.3, $w_{5}$ = 1.7, $w_{6}$ = 1.4, $w_{7}$ = 0.9, $w_{8}$ = 0.4, $R_{1}$ = 6.2, $l_{1}$ = 11.6, $l_{2}$ = 11.9, $l_{3}$ = 16.5, $W_{0}$ = 11.4, *P* = 12, $d_{1}$ = 13.6 (unit: mm).

**Figure 6:**
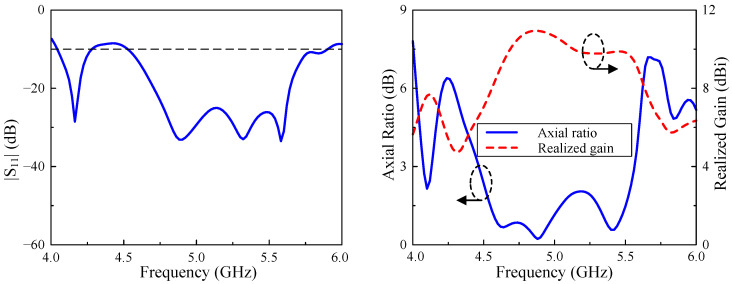
Simulated performances of the proposed antenna.

The simulated performances of the proposed array are shown in Figure 6. In comparison with the single element described in the previous section, the $|S_{11}|$ BW is improved from 16.2% (4.75–5.59 GHz) to 27.6% (4.52–5.90 GHz). Similarly, the AR BW is also increased from 2.6% (4.82–4.96 GHz) to 22.0% (4.46–5.56 GHz). Regarding the broadside gain, the array exhibits better broadside gain, which is 10.9 dBi compared to 6.8 dBi.

**Figure 7:**
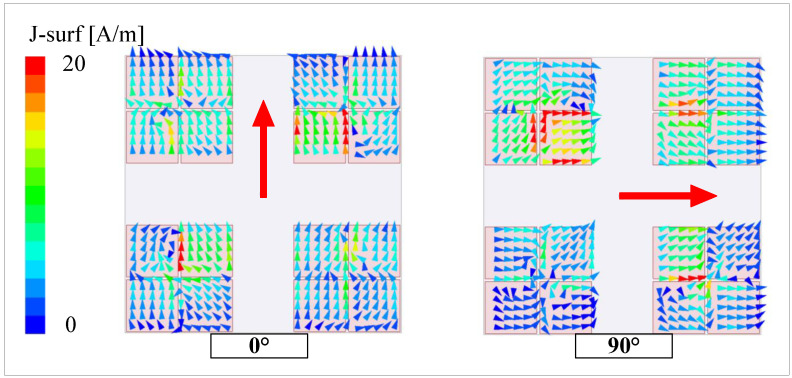
Simulated surface current distribution of the proposed antenna on the MS structure at 5.0 GHz.

The CP operation principle of the proposed antenna configuration can be explained by observing the surface current distribution diagram, as shown for the frequency of 5.0 GHz in Figure 7. Here, the current distribution on the rotated patches is not shown for the sake of brevity, since the primary radiating elements responsible for generating CP waves are the MS structures. It is noticeable that when the input signal phase is shifted by 90°, the direction of the current distribution vectors are likely to rotate in clockwise. It is to claim that the antenna works in LHCP mode.

## 4. Design Optimization

The optimization process has been implemented via a number of stages to determine the best values of antenna dimensions. The key considerations in this paper are the operating bandwidth, the AR bandwidth, and the broadside gains.

### 4.1. Operating Band Optimization

**Figure 8:**
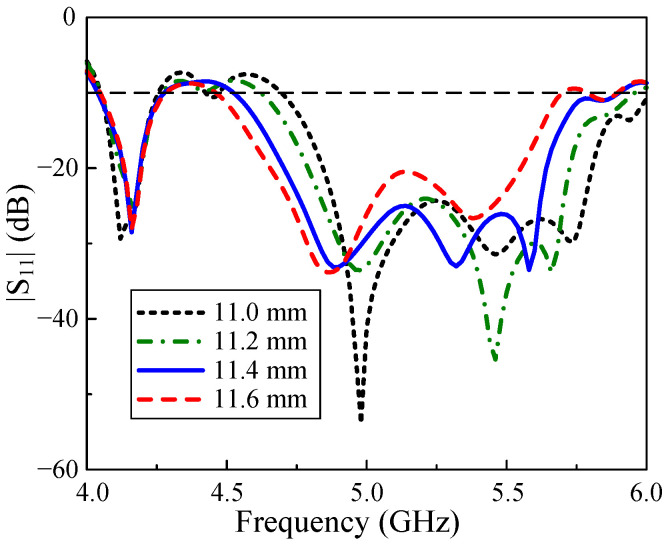
Simulated $|S_{11}|$ of the proposed array with different variations of $W_{0}$.

**Figure 9:**
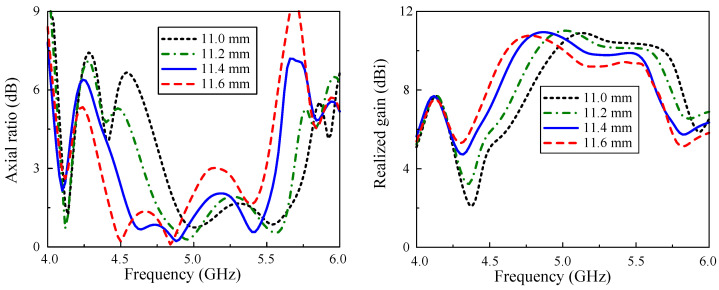
Simulated AR and realized gain of the proposed array with different values of $W_{0}$.

It has been validated by simulation that the unit-cell width $W_{0}$ poses the most noticeable impact on the operating band of the proposed array. This is due to the fact that the MS is the primary radiating element and its size will definitely affect the resonant frequency. The simulated results of reflection coefficients when varying $W_{0}$ is shown in Figure 8. Overall, the −10 dB BWs of all cases are quite similar. However, an observation on the figure indicates that the operating band of the antenna is more likely to be shifted to a lower frequency when increasing the unit-cell sizes. Regarding far-field performances, the simulated results on the axial ratio and realized gain are demonstrated in Figure 9. It is obvious that the changes in broadside gains and axial ratios of the antenna show similar tendencies to the corresponding cases of $|S_{11}|$.

### 4.2. AR Optimization

**Figure 10:**
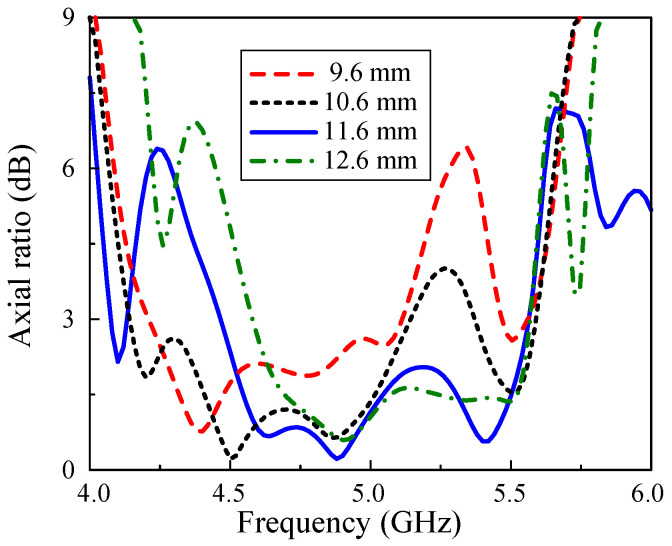
Simulated AR of the proposed array with the variations of $l_{1}$.

As the Y-shaped patch is the CP source to excite the MS layer, the AR performance depends critically on the length of this patch, $l_{1}$, whose simulation results are displayed in Figure 10. Obviously, all of the investigated cases offer a noticeably broad frequency range that have axial ratios of lower than 3 dB. With $l_{1}$ = 9.6 mm and 10.6 mm, the AR BWs of the array are 18.0% and 20.2%, respectively. The drawbacks of these variations include the narrower bandwidths compared to the selected length and small perturbation ranges around 5.3 GHz. Furthermore, the highest $l_{1}$ of 12.6 mm achieves the upper frequency of the 3 dB AR BW at 5.56 GHz, which is similar to that of the optimized parameter of 11.6 mm. However, the lower frequency is 4.58 GHz, resulting in a smaller AR BW when compared to the latter variation.

### 4.3. Realized Gain Optimization

**Figure 11:**
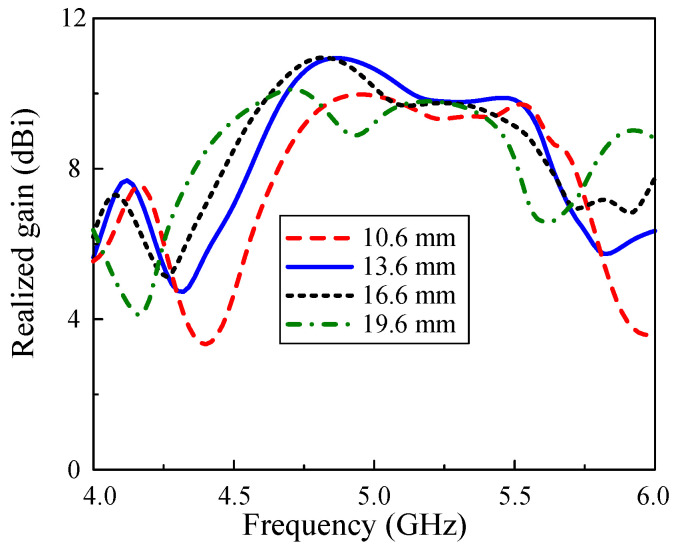
Simulated realized gain of the proposed array with the variations of $d_{1}$.

Finally, the study of how to control the broadside gain of the antenna array concludes that the edge-to-edge distance between each MS structure offers the most significant effect. According to the investigation in [19,25], the optimal value for the center-to-center element spacing is from 0.7λ to 1.0λ. Here, the element spacing is studied within this range to choose the optimized gain value. Figure 11 describes the simulated gain results of the proposed array when the space between each MS network $d_{1}$ varies. The highest and lowest investigated values of MS-structure distance offer the gain curves of less than 10 dBi generally. With $d_{1}$ = 16.6 mm, the broadside gain performance shows the similar shape to that of the optimized case. Nevertheless, the gain from the frequency of 5.0 GHz of the former case is worse than the latter one by a minor gap, which is why the best value of $d_{1}$ is chosen at 13.6 mm.

## 5. Measured Results

**Figure 12:**
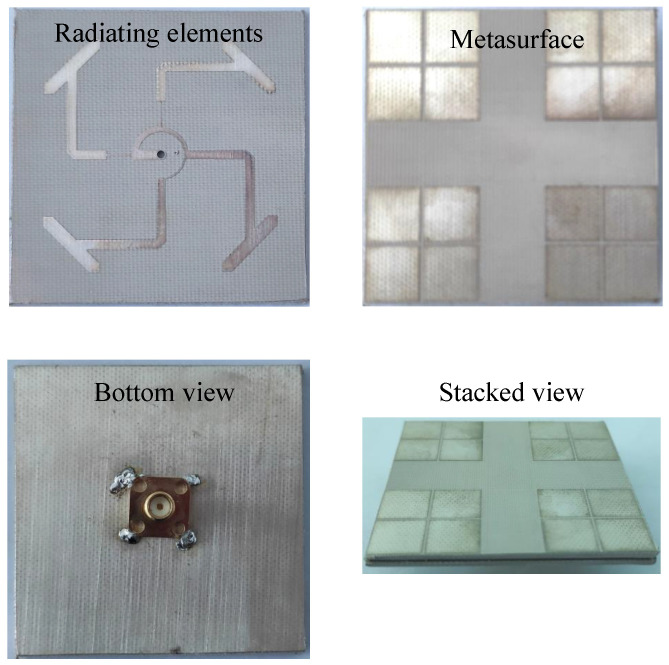
Photographs of the fabricated antenna array.

In order to validate the feasibility of the proposed array, an antenna prototype has been fabricated, as depicted in Figure 12. The antenna array is printed on two sheets of Taconic TLY-5 dielectric substrate. A 50 Ω SMA connector is occupied to feed the antenna at the center of the patch. The overall dimensions of the antenna are 1.02 $\lambda_{0}$ × 1.02 $\lambda_{0}$ × 0.05 $\lambda_{0}$, where $\lambda_{0}$ is the free space wavelength at the center frequency of 5.0 GHz. For measurement, the reflection coefficient is tested by the Keysight PNA-X Network Analyzer N5242A manufactured by Keysight Technologies, Inc. (Santa Rosa, CA, USA). Meanwhile, the far-field parameters are carried out in an isolated anechoic chamber. The proposed antenna functions as the receiver, whereas a conventional wideband horn antenna serves as the transmitter. During the measurement, the receiving antenna is rotated at 360° azimuth range to achieve the signal strength at every orientations. These data are then further analyzed to extract far-field radiation patterns of the tested antenna. In fact, the difference between the simulations and measurements always exists, which mostly comes from the tolerance in fabrication. Meanwhile, unideal in measurement setup is also another possible reason.

**Figure 13:**
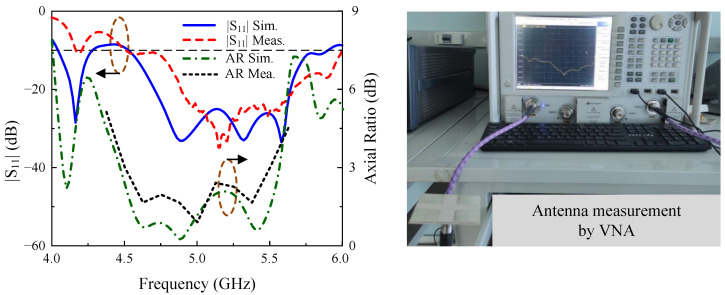
Simulated and measured $|S_{11}|$ and axial ratio of the proposed antenna.

The simulated and measured reflection coefficient results of the fabricated prototype are illustrated in Figure 13. Overall, there is a good agreement between the simulation and measurement. Some disparities can be attributed to the fabrication tolerances and deficiency while setting up the measurement environment. The measured $|S_{11}|$ is from 4.50 to 5.99 GHz, which is equivalent to 29.8%.

**Figure 14:**
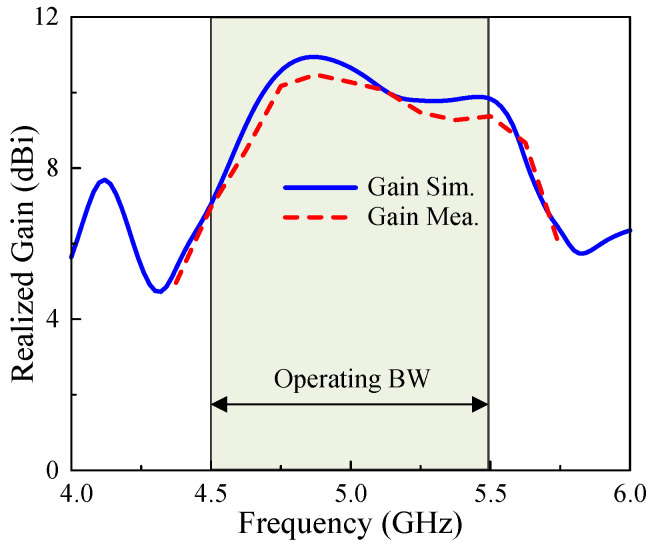
Simulated and measured broadside gain results of the proposed antenna.

The far-field performances in terms or AR and gain are presented in Figure 14. The measured AR BW is 20%, from 4.50 to 5.50 GHz. Additionally, the SP-fed MS-based array provides a gain of better than 7 dBi within the operating BW and a peak gain of 10.5 dBi. The peak gain has an enhancement of about 4 dBi while comparing the single element and the array configurations. Regarding the antenna efficiency, the simulated value is about 90%. In measurement, the antenna efficiency was not measured due to the limit of the chamber; however, it can still be estimated based on the realized gain measurements. Here, the gain difference between simulation and measurement is about 0.6 dBi. Thus, the calculated measured efficiency is about 80%.

**Figure 15:**
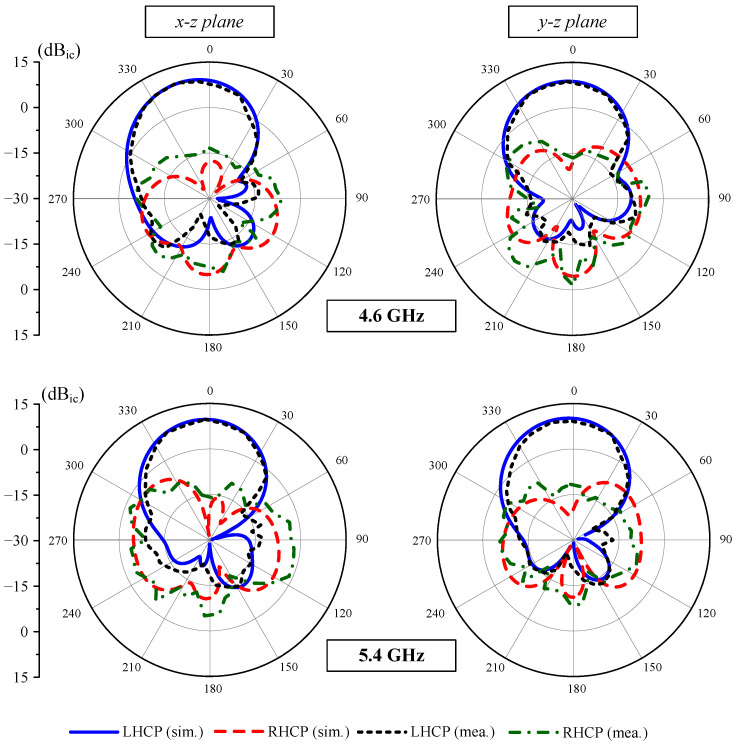
Simulated and measured radiation patterns of the proposed array at 4.6 GHz and 5.4 GHz.

The radiation patterns of the proposed antenna at 4.6 GHz and 5.4 GHz are depicted in Figure 15. Obviously, the SP-fed MS-based array produces an LHCP mode toward the +z-direction. The polarization isolation at the broadside direction is relatively high, which is always greater than 19 dB. In addition, a high front-to-back ratio is another advantage of this configuration, which is better than 20 dB across the operating BW.

## 6. Comparison

Table 1 compares the proposed prototype and the other related CP printed antennas. It is evident that all of the reported techniques have effectively overcome the BW limitation on conventional patch antennas. Although the highest peak gain could be seen in the prototype of [12] at 18.17 dBi, the trade-off of this antenna is a very large-scale configuration. The designs in [13,16] produced the smallest profiles, but their peak gains are much lower than 10 dBi. Most of the compared items in this table offer high peak gains of better than 10 dBi, while their drawback is large overall dimensions of larger than 1.2 $\lambda_{0}$. To sum up, some of the published works can achieve wideband and high gain operation; however, their big size is the critical disadvantage. Meanwhile, several compact designs perform narrowband and low gain radiation. The proposed design shows a good trade-off between the size and operation characteristics by using a compact CP element. It is the main contribution of the presented work.

## 7. Conclusions

This paper presents a design of a microstrip patch antenna that provides broadband CP operation and relatively high broadside gains. The antenna prototype is fabricated for verification, in which simulated and measured results show good agreement. The impedance BW of the antenna array is 29.8% and the AR BW is 20.0%. The antenna has a peak gain of 10.5 dBi, while the broadside gain within the operating frequency range is consistently better than 7.1 dBi. In comparison with the related works, the proposed design has the benefits of a wide operating band and considerable overall gains with a compact dimension. The noticeable performance of this design makes it a highly potential candidate to be applied in C-band satellite communication systems.

## Figures and Tables

**Table 1:** Performance comparison among broadband CP patch antennas.

Ref.	Method	Dimensions ($\lambda_{0}$)	Impedance BW (%)	AR BW (%)	Peak Gain (dBic)
[12]	Parasitic element	4.5 × 4.9 × 0.06	29.43	27.36	18.17
[13]	Parasitic element	1.02 × 1.02 × 0.028	25.8	20.6	8.0
[14]	Parasitic element, stacked patch	1.02 × 0.86 × 0.13	32.0	22.8	8.5
[15]	Sequential-phase feed, metasurfaces	1.22 × 1.22 × 0.15	64.0	28.2	6.3
[16]	Sequential-phase feed, irregular ground (DGS)	1.43 × 1.43 × 0.01	90.9	42.4	7.17
[17]	Sequential-phase feed	1.85 × 1.90 × 0.03	74.0	49.5	10.2
[18]	Sequential-phase feed	1.2 × 1.2 × 0.04	42.9	36.4	11.5
[19]	Sequential-phase feed	2.2 × 2.2 × 0.31	93.81	22.64	12.32
				49.16	11.66
[20]	Sequential-phase feed, 2 suspended metal rods	1.6 × 1.6 × 0.154	67.8	49.7	13.1
[21]	Sequential-phase feed, parasitic elements	1.7 × 1.7 × 0.06	37.7	28.0	15.8
[22]	Sequential-phase feed, stacked patch	0.92 × 0.92 × 0.073	33.6	19.4	10.2
Prop.	Sequential-phase feed, metasurface	1.02 × 1.02 × 0.05	29.8	20.0	10.5

## Data Availability

The original contributions presented in the study are included in the article, further inquiries can be directed to the corresponding author.
